# Double Dissociation of Amygdala and Hippocampal Contributions to Trace and Delay Fear Conditioning

**DOI:** 10.1371/journal.pone.0015982

**Published:** 2011-01-19

**Authors:** Jonathan D. Raybuck, K. Matthew Lattal

**Affiliations:** Department of Behavioral Neuroscience, Oregon Health and Science University, Portland, Oregon, United States of America; Georgia Health Sciences University, United States of America

## Abstract

A key finding in studies of the neurobiology of learning memory is that the amygdala is critically involved in Pavlovian fear conditioning. This is well established in delay-cued and contextual fear conditioning; however, surprisingly little is known of the role of the amygdala in trace conditioning. Trace fear conditioning, in which the CS and US are separated in time by a trace interval, requires the hippocampus and prefrontal cortex. It is possible that recruitment of cortical structures by trace conditioning alters the role of the amygdala compared to delay fear conditioning, where the CS and US overlap. To investigate this, we inactivated the amygdala of male C57BL/6 mice with GABA _A_ agonist muscimol prior to 2-pairing trace or delay fear conditioning. Amygdala inactivation produced deficits in contextual and delay conditioning, but had no effect on trace conditioning. As controls, we demonstrate that dorsal hippocampal inactivation produced deficits in trace and contextual, but not delay fear conditioning. Further, pre- and post-training amygdala inactivation disrupted the contextual but the not cued component of trace conditioning, as did muscimol infusion prior to 1- or 4-pairing trace conditioning. These findings demonstrate that insertion of a temporal gap between the CS and US can generate amygdala-independent fear conditioning. We discuss the implications of this surprising finding for current models of the neural circuitry involved in fear conditioning.

## Introduction

A key finding from studies of Pavlovian fear conditioning is that amygdala function is required to associate neutral conditioned stimuli (CSs) with aversive events (unconditioned stimuli; USs). This has been demonstrated in many fear conditioning situations where the neutral stimulus is a discrete stimulus or a physical context. Although there are different theories for the role of the amygdala in this conditioning process (e.g. [1]–[2]), there is general agreement that the amygdala is involved in some aspect of fear learning. These models have revealed a great deal about the biochemical, genetic, and epigenetic mechanisms involved in the circuitry that underlies fear learning and memory (e.g. [3]–[6]). However, within this circuitry, there are a number of caveats about the contribution of various structures to different components of this task.

When a CS is paired with a US, typically the US occurs coincident with or immediately after CS offset. This delay-cued fear conditioning results in a strong CS-US association that depends on plasticity largely thought to occur in the amygdala [2]. Insertion of an interval (the trace interval) between the CS and US recruits the dorsal hippocampus (DH), prelimbic cortex (PrL), anterior cingulate cortex, and entorhinal and perirhinal cortices to this trace-cued fear conditioning [7]–[12]. Surprisingly little is known about the contributions of the amygdala to trace fear conditioning [13]. Given the well-documented importance of the amygdala in fear learning, one should expect it to also be critical for trace fear conditioning [13]. However, because of the additional circuitry recruited by trace fear conditioning, it is possible that the amygdala is less critical for the acquisition or consolidation of the trace fear memory. Although the nature of the interaction between the hippocampus and amygdala in contextual fear conditioning is well studied, it is not yet understood how these regions interact to support trace conditioning. One possibility is that the hippocampus maintains a representation of the CS during the trace interval and interacts with cortical regions to assign salience and predictive value to that representation. However, it is not yet clear whether amygdala activity is a critical component of this task. It may be that unlike delay and contextual conditioning, activity in the hippocampus and cortex are sufficient to support acquisition of trace fear conditioning.

To investigate the involvement of the dorsal hippocampus and amygdala in trace fear conditioning, we temporarily inactivated these regions via intra-cranial infusion of muscimol, a GABA_A_ receptor agonist, prior to trace or delay fear conditioning. To further examine amygdala contributions to trace and contextual conditioning, muscimol was infused before or after conditioning and before different trace fear conditioning protocols.

## Methods

### Subjects

These studies used 159, 8–12 week old, male C57Bl6/J mice, individually housed in standard colony cages, maintained on a 12/12 hour light/dark cycle. All animals received *ad lib* food and water, and all studies were authorized by the Oregon Health & Science University Institutional Animal Care and Use Committee (Protocol ID B11039) and conducted in accordance with the ethical guidelines of the Society for Neuroscience and the National Institutes of Health.

### Drugs

Muscimol (Sigma-Aldrich, St. Louis, MO) in 0.25 ul PBS, was infused at 0, 0.25, or 0.5 ug/side at a rate of 0.25 µl/min through stainless steel cannulae into the DH (26 g) or amygdala (33 g). The infusion cannulae were attached with PE50 to Hamilton syringes (Hamilton, Reno, NV) controlled by a microinfusion pump. Injection cannulae were left in place for 30 s after infusion.

### Apparatus

Training and testing for contextual conditioning was conducted in 21.5 cm circular plexiglas chambers described in [14]. An 85 dB CS was administered through a sound generator (Coulbourn Instruments, Whitehall, PA), and a 0.35 mA footshock US was administered through the rod floor with a shock scrambler/generator (Coulbourn). To provide a distinct olfactory cue the apparatus was wiped down with 0.1% acetic acid prior to conditioning or testing sessions. Training and context testing sessions were controlled by an IBM-PC running Graphic State software (Coulbourn). Testing for cued conditioning was conducted in rectangular conditioning chambers (Med-Associates, St. Albans, VT) with white Plexiglas floors, situated in sound attenuating cubicles. These chambers were located in a room different from the conditioning room. CS was generated with ANL-926 (Med) and administered through speakers mounted on the left wall of the chambers, controlled by an IBM-PC running MED-PC 4 (Med). The altered context was cleaned with 70% ethanol. In both contexts ventilation fans provided 65dB background noise.

### Surgical Procedures

Mice were anesthetized with isoflurane (2%–5%), mounted in a stereotaxic apparatus (David Kopf Instruments, Tujunga, CA), and the scalp was scrubbed and excised to expose the skull. Holes were drilled for anterior and lateral coordinates; guide cannulae were lowered into place with the stereotaxic, to match D/V coordinates; and permanently secured with dental cement [15]. DH coordinates were A/P -1.7, M/L 1.5, D/V -2.3 mm and amygdala coordinates were A/P -1.46, M/L 3.1, D/V -4.8 mm from bregma [16]. Stainless steel stylets were inserted into the cannulae to maintain patency during the 5 day post-surgical recovery period.

### Trace fear conditioning

Separate experiments examined the effects of 1, 2, or 4 trace conditioning trials. Each session began with activation of a house light, 2 minutes after which a 30 s CS was activated, followed 30 s later by a 2 s US presentation [17]. In the two- and four-trial experiments, each CS-US presentation was separated by a variable 90 s inter-trial interval (ITI). After the final trial, mice remained in the chambers for an additional 30 s, after which the houselight was inactivated and mice were returned to their home cages. The session lengths were 3.5, 6.5, and 11.5 min for the one-, two-, and four-trial experiments, respectively.

### Delay fear conditioning

Delay fear conditioning was similar to trace fear conditioning, except that the US was presented during the final 2 s of the CS. Mice received two CS-US pairings in a 6.5 min session.

### Testing

To test for contextual learning, 24 hr following training, mice were placed in the training apparatus, the house light was activated and freezing was assessed for 5 min. To test for cued learning, 48 hr following training mice were placed in the cued-testing apparatus and assessed for generalized freezing for 3 min, followed by two 3 min-long CS presentations separated by a 3 min ITI, followed with a 3 min post-CS period. Freezing was assessed across the entire session, and for analysis the 3 minute, Pre-CS, ITI, and Post-CS periods were combined and reported as “Altered” and the 2 CS presentations were combined and reported as “CS”. Freezing was defined as the absence of all movement except respiration [18], assessed for one second at ten second intervals by an observer unaware of treatment assignments and reported as percent freezing [19].

### Histology

**Figure 1:**
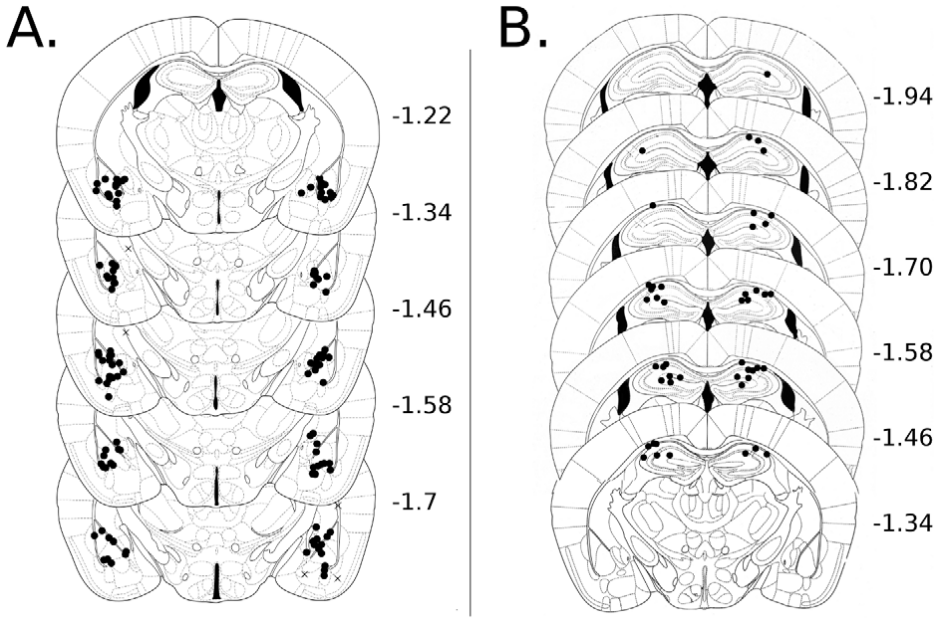
Schematic representation of DH and amygdala cannulae placements. Placements determined to be outside of the target regions are marked with crosses, placements within the target region are marked with solid circles.

All brains were post-fixed in 4% paraformaldehyde in PBS for at least 24 hours, sliced on a cryostat, mounted and nissl stained with cresyl-violet. Infusion sites were confirmed by observing gliosis along the infusion cannula tracts. All dorsal hippocampal infusions were within target regions and all but three amygdala infusions were within or adjacent to the amygdala, Figure 1. Missed placements were excluded from analysis.

### Analysis

Analysis was conducted with RExcel 3.1.1 [20]. Data were analyzed with one-way ANOVA followed with Tukey's post-hoc tests. All data are reported as mean ± the SEM, significant differences from vehicle were defined as p<0.05.

## Results

### Dorsal Hippocampal Inactivation Produces Deficits in Contextual But Not Delay Fear Conditioning (Figure 2A)

**Figure 2:**
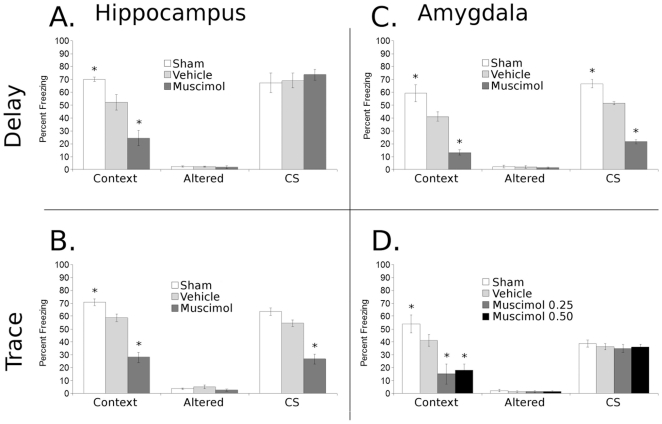
Effects of DH or amygdala inactivation on contextual and cued components of delay and trace fear conditioning. Infusion of muscimol into the DH disrupts acquisition/consolidation of trace (**B**) and contextual (**A & B**) but not delay conditioning (**B**), while muscimol infusion into the amygdala disrupts delay (**C**) and contextual (**C & D**), but not trace conditioning (**D**). These results suggest that the amygdala may be differently involved in trace conditioning than in contextual and delay conditioning. Additionally, deficits in vehicle compared to sham in contextual (**A, B, C & D**) and CS freezing (**B & C**) show that disruption of DH or amygdala can interfere with conditioning. Subjects per group from top to bottom by panel, were: (**A**) 4, 6, 6; (**B**) 4, 6, 6; (**C**) 8, 8, 8; (**D**) 7, 14, 6, 12. Data represented as Mean ± SEM, * denotes significant difference from vehicle control group.

Infusion into the DH prior to 2-pairing delay conditioning disrupted contextual fear conditioning [F(2,13) = 19.1, p<0.005] but not Altered or CS freezing. Post-hoc analysis of contextual freezing demonstrated that vehicle-treated mice froze significantly less than sham-treated controls, and muscimol-treated mice froze less to context exposure than vehicle-treated mice or sham controls (Figure 2A). These results demonstrate that perturbation of the DH by vehicle infusion produces small deficits in contextual learning during delay fear conditioning, and that inactivation of this brain region with muscimol produces large deficits in contextual learning, confirming that the DH is critical to contextual but not delay fear conditioning.

### Dorsal Hippocampal Inactivation Produces Deficits in Contextual and Trace Fear Conditioning (Figure 2B)

Infusion into the DH prior to 2-pairing trace conditioning affected contextual [F(2,21) = 52.0, p<0.005] and CS [F(2,21) = 41.7, p<0.005], but not Altered freezing (Figure 2B). Post-hoc analysis revealed that in both contextual and CS freezing muscimol infused mice froze significantly less than vehicle controls, and that vehicle infusion significantly impaired contextual learning compared to sham controls, while a trend toward a deficit was observed in CS freezing. These results demonstrate that vehicle infusion into the DH disrupts contextual learning, and that inactivation of the DH with muscimol disrupts both contextual and trace-cued fear learning compared to vehicle controls.

### Amygdala Inactivation Produces Deficits in Contextual and Delay Fear Conditioning (Figure 2C)

Infusion into the amygdala prior to delay fear conditioning affected contextual [F(2,13) = 56.1, p<0.005] and CS [F(2,13) = 148.6, p<0.005], but not Altered freezing (Figure 2C). Post-hoc analysis showed that inactivation of the amygdala produced deficits in contextual and CS freezing compared to vehicle or sham controls, and that vehicle infusion produced deficits in CS freezing compared to sham controls, confirming that the amygdala is critically involved in acquisition of contextual and delay fear learning.

### Amygdala Inactivation has No Effect on Trace Fear Conditioning (Figure 2D)

Infusion into the amygdala before 2-pairing trace fear conditioning affected contextual [F(3,35) = 8.1, p<0.005], but not CS or Altered freezing (Figure 2D). Post hoc analysis revealed that muscimol (0.25 or 0.50 ug/side) produced deficits in contextual freezing compared to vehicle controls, suggesting that amygdala activity is not required for trace fear conditioning.

### Amygdala Inactivation Effects in Trace Conditioning Are Independent of Training Protocol (Figure 3A, B)

**Figure 3:**
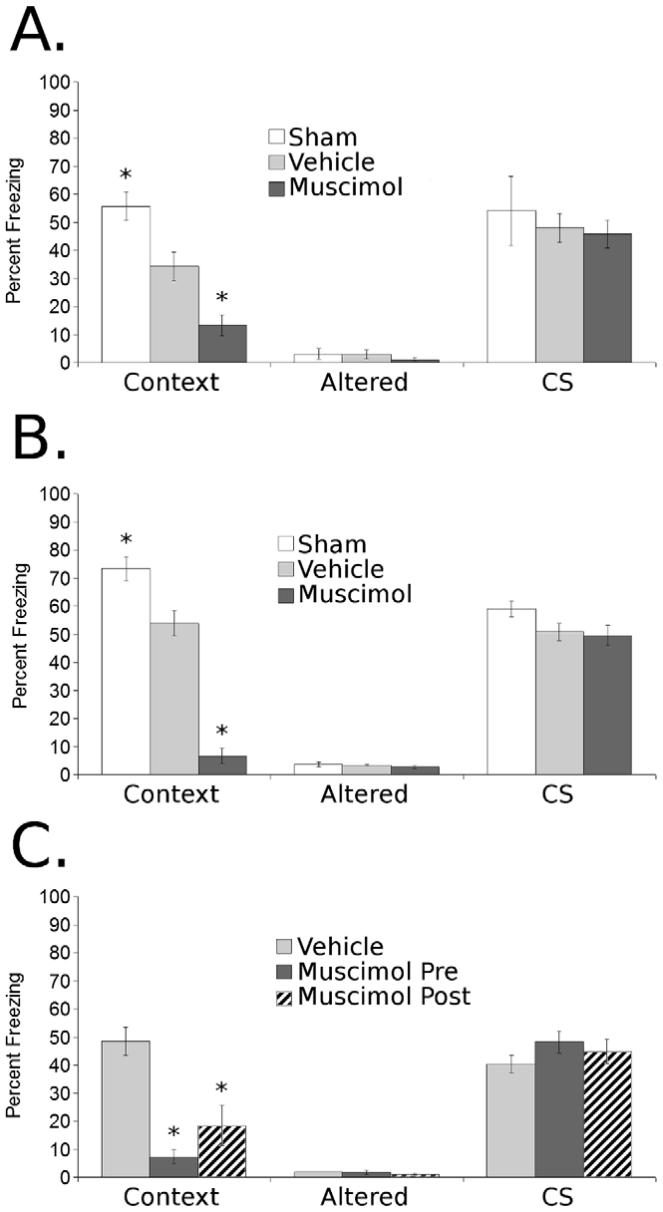
Parametric manipulations demonstrate that amygdala inactivation has similar effects on 1-pairing and 4-pairing trace fear conditioning, and that amygdala inactivation prior to or immediately after training of 2-pairing trace conditioning has similar effects. (**A**) Inactivation of the amygdala prior to 1-pairing or (**B**) 4-pairing trace fear conditioning produces deficits in the contextual component but does not affect the cued component or freezing to an altered context. Additionally, comparison of sham and vehicle groups demonstrates that infusion of vehicle into the amygdala produces deficits in contextual but not trace conditioning and does not affect freezing to an altered context. (**C**) Inactivation of the amygdala 20-minutes pre-training or immediately post-training produced deficits in the contextual but not the cued component of 2-pairing trace fear conditioning, and did not affect freezing to an altered context. Subjects per group from top to bottom by panel, were: **A** 4, 6, 6; **B** 4, 4, 5; **C** 4, 6, 6. Data represented as Mean ± SEM, * denotes significant difference from vehicle control group.

**Figure 4:**
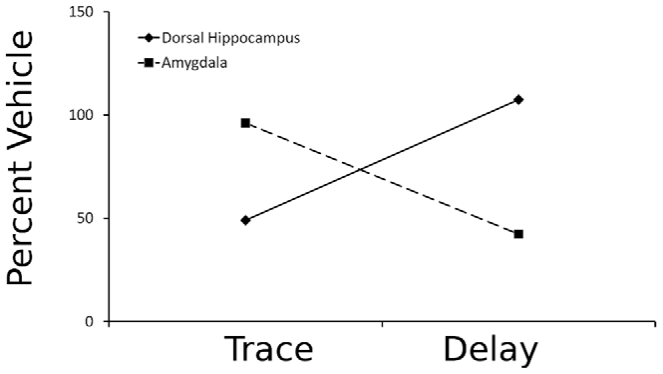
Double dissociation of effects of muscimol infusion on 2-pairing trace and delay cued freezing. Inactivation of the DH with muscimol produces deficits in trace but not delay conditioned CS freezing, while inactivation of the amygdala produces deficits in delay but not trace conditioned CS freezing. These findings suggest that amygdala independent circuitry can support trace fear conditioning. Data are Muscimol (0.25 ug/side) freezing to CS from experiments 1–4 as percent vehicle.

To determine if the lack of effect of amygdala inactivation on trace fear conditioning was particular to a 2-pairing trace fear conditioning protocol, mice received 1-pairing (Figure 3A) or 4-pairing (Figure 3B) trace fear conditioning. There were significant effects of infusion on contextual conditioning in both 1-pairing [F(2,13) = 23.4, p<0.005] and 4-pairing [F(2,10) = 90.4, p<0.005] but no effects on CS or Altered freezing in either task. Post-hoc analysis showed that in both 1-pairing and 4-pairing experiments muscimol infusion prior to training produced deficits in contextual freezing compared to vehicle or sham treated groups, and that vehicle infusion produced deficits compared to sham controls. These results suggest that the effects of amygdala inactivation on fear conditioning generalize across multiple protocols.

### Amygdala Inactivation Pre- or Post-Training Produces Deficits in Contextual But Not Trace Fear Conditioning (Figure 3C)

To determine if the effects of muscimol infusion into the amygdala on contextual learning were due to interference with processes related to acquisition, such as locomotor activity during training, muscimol or vehicle was infused into the amygdala 20 min pre- or immediately post-training (Figure 3C). In this study, all groups received muscimol or vehicle infusion both pre- and post-training, to equate handling conditioning between groups [21]. Thus the “vehicle” *g*roup received two vehicle infusions, the “pre-training” group received muscimol pre- and vehicle post-training, and the “post-training” group received vehicle pre- and muscimol post-training. Muscimol infusion pre- or post-training produced deficits in contextual learning [F(2,16) = 15.9, p<0.005], but had no effect on CS or Altered freezing. Post-hoc tests showed that mice receiving pre- or post-training muscimol froze significantly less to the context than vehicle controls.

## Discussion

The key finding from our results is that inactivation of the amygdala did not affect acquisition or consolidation of trace fear conditioning, even though delay and contextual fear conditioning were impaired. Further, inactivation of the DH impaired trace and contextual conditioning, but did not affect delay conditioning. Together, these findings suggest that insertion a temporal gap between the CS and the US allows amygdala-independent fear conditioning to occur. The dissociation between hippocampal and amygdala involvement in trace and delay fear conditioning in our experiments is evident in Figure 4, which summarizes these key findings.

The absence of an effect of amygdala inactivation on trace fear conditioning in the present findings is surprising, but it is unlikely to be explained by insensitive experimental manipulations. Animals that did not show trace fear conditioning deficits showed deficits in contextual fear conditioning and the same muscimol injections resulted in robust deficits in delay fear conditioning. Further, parametric control experiments demonstrate that the lack of effect of amygdala inactivation on trace fear conditioning is not due to under- or over-training as amygdala inactivation had no effect with one, two, or four CS-US pairings. Finally, the finding that muscimol injections into the DH disrupted trace fear conditioning demonstrates that the behavioral parameters in this paradigm were sensitive to disruption by neurobiological manipulations. Thus, the absence of an effect of amygdala inactivation on trace fear conditioning is strengthened by the presence of effects of amygdala inactivation on contextual and delayed fear conditioning, as well as by the effects of hippocampal inactivation on trace fear conditioning.

Our amygdala infusion cannulae were targeted toward the basolateral nucleus (BLA) of the amygdala, which is critically involved in contextual fear conditioning [22]–[23], whereas the lateral amygdala (LA) supports auditory processing in fear conditioning but not necessarily contextual learning [24]–[25]. Thus, one possible explanation for our finding that amygdala inactivation disrupted contextual but not trace fear conditioning could be that our infusions inactivated the BLA but not the LA, and the LA supports trace conditioning. However, we demonstrate that inactivation of the amygdala with muscimol produces deficits in delay as well as contextual fear conditioning. Thus, our infusions are sufficient to affect delay fear conditioning, either through LA inactivation or because the BLA plays a critical role in delay conditioning. This makes it unlikely that our results can be explained by insufficient amygdala inactivation by muscimol infusion. Additionally estimates suggest that muscimol diffusion with this procedure is approximately 1 mm^3^ [26]–[27], suggesting that our infusions were sufficient to inactivate the entire amygdala. While such diffusion precludes conclusions about which amygdala nuclei were inactivated in these studies, the pattern of results clearly suggests that the amygdala plays a different role in trace than in delay and contextual conditioning.

In these studies the GABA_A_ receptor agonist muscimol was used to inactivate the DH and amygdala. As muscimol is selective to GABA_A_ receptors it is possible that our results demonstrate distinct roles for GABA_A_ receptor expressing neurons in the amygdala in trace and delay fear conditioning. Additionally, it should be noted that muscimol temporarily inhibits neural activity, and it is possible that plasticity supporting learning could occur independent of local neural activity. Whether these findings are because of distinct GABA receptor involvement in these forms of learning or distinct regional involvement in these tasks will require further study.

Previous studies have shown that amygdala-independent contextual fear conditioning can occur with intensive training [28]–[29], suggesting that it is possible under unique training conditions for amygdala-independent fear conditioning to occur. One view of amygdala function in fear conditioning is that rather than acting as the seat of plasticity, it plays a modulatory role increasing the strength of thalamo-cortical associations between CS and US [30]–[31]. It may be that extensive training establishes CS-US associations through pathways independent of the amygdala, and that trace conditioning facilitates learning through these circuits. However, it is also possible that the amygdala is engaged by trace conditioning [9], [13], but amygdala activity is not necessary for associative learning. Future work will need to determine the precise role that the amygdala plays in the trace circuit.

### Alternate Pathways to Trace Conditioning

Brain regions involved in trace fear conditioning are highly interconnected. Notably, the ventral hippocampus, thought to act as a conduit between the DH and amygdala [32], has neurons that extend projections to both the amygdala and PrL [33]. Additionally, the ventral hippocampus mediates connectivity between the entorhinal/perirhinal cortices and the amygdala [34]. Thus, it could be that activity in the hippocampus and cortex can support acquisition of trace conditioning independent of the amygdala, and generate responses to the CS by hijacking amygdala output through ventral hippocampal projections to the amygdala. However, the PrL is also important form trace conditioning [35]–[37], [12] and its connectivity suggests that it could play a more direct role.

Stimulation of the PrL increases freezing in rats [38], suggesting that the PrL positively modulates fear responses. Additionally, neuronal activity in the PrL during the CS increases during trace fear conditioning [39], thus it may be critically involved in generating fear responses to the trace-conditioned CS. However, the pathways through which these responses occur are not yet known. PrL connectivity suggests multiple routes through which it could directly initiate fear responses. PrL projections to the periaquaductal gray could bypass the amygdala entirely allowing the PrL to directly evoke fear responses [40]–[41]. An additional route through which the PrL could generate fear responses is through projections to the intercalated-cell masses of the amygdala. These cells modulates inhibition of the CeA [42], which is involved in fear response. Thus, PrL projections to the ITCm could allow the PrL to hijack amygdala output, controlling fear expression and subjugating the amygdala to an output structure rather than a center for CS-US association.

Clearly there are multiple mechanisms that may support trace fear conditioning. Our findings demonstrate that inserting a temporal gap between the CS and US may allow fear memories to form independent of amygdala activity, although it is not yet clear whether plasticity in the amygdala supports this task. These findings add a level of complexity to current thinking about the circuitry underlying fear learning and add to a body of literature that shows that trace fear conditioning is supported by circuitry distinct from that of delay and contextual conditioning.
